# Tumour surface regularity predicts survival and benefit from gross total resection in IDH-wildtype glioblastoma patients

**DOI:** 10.1186/s13244-025-01900-2

**Published:** 2025-02-17

**Authors:** Peng Lin, Jin-Shu Pang, Ya-Dan Lin, Qiong Qin, Jia-Yi Lv, Gui-Qian Zhou, Tian-Ming Tan, Wei-Jia Mo, Gang Chen

**Affiliations:** 1 Department of Medical Ultrasound, Fujian Medical University Union Hospital, Fuzhou, China; 2 Department of Medical Ultrasound, The First Affiliated Hospital of Guangxi Medical University, Nanning, China; 3 Department of Medical Image, The Third People’s Hospital of Ganzhou, Ganzhou, China; 4 Department of Pathology, The First Affiliated Hospital of Guangxi Medical University, Nanning, China

**Keywords:** Glioblastoma, MRI, Sphericity, Extent of resection

## Abstract

**Objectives:**

To evaluate the ability of sphericity in glioblastomas (GBMs) for predicting overall survival (OS) and the survival benefit from gross tumour resection (GTR).

**Methods:**

Preoperative MRI scans were retrospectively analysed in IDH-wildtype GBM patients from two datasets. After MRI preprocessing and tumour segmentation, tumour sphericity was calculated based on the tumour core region. The prognostic value of tumour surface regularity was evaluated via Kaplan–Meier (K-M) plots, univariate and multivariate Cox proportional hazards analyses. In different surface regularity subgroups, the OS benefit from GTR was evaluated via K-M plots and the restricted mean survival time (RMST).

**Results:**

This study included 367 patients (median age, 62.0 years [IQR, 54.5–70.5 years]) in the discovery cohort and 475 patients (median age, 63.6 years [IQR, 56.2–71.3 years]) in the validation cohort. Sphericity was an independent predictor of OS in the discovery (*p* = 0.022, hazard ratio (HR) = 1.45, 95% confidence interval (CI) 1.06–1.99) and validation groups (*p* = 0.007, HR = 1.38, 95% CI: 1.09–1.74) according to multivariate analysis. Age, extent of resection, and surface regularity composed a prognostic model that separated patients into subgroups with distinct prognoses. Patients in the surface-irregular subgroup benefited from GTR, but patients in the surface-regular subgroup did not in the discovery (*p* < 0.001 vs. *p* = 0.056) and validation datasets (*p* < 0.001 vs. *p* = 0.11).

**Conclusions:**

The high surface regularity of IDH-wildtype GBM is significantly correlated with better OS and does not benefit substantially from GTR.

**Critical relevance statement:**

The proposed imaging marker has the potential to increase the survival prediction efficacy for IDH-wildtype glioblastomas (GBMs), offering a valuable indicator for clinical decision-making.

**Key Points:**

- Sphericity is an independent prognostic factor in IDH-wildtype glioblastomas (GBMs). High sphericity in IDH-wildtype GBM is significantly correlated with better survival.
- GBM patients with low sphericity could receive survival benefits from gross tumour resection.

**Graphical Abstract:**

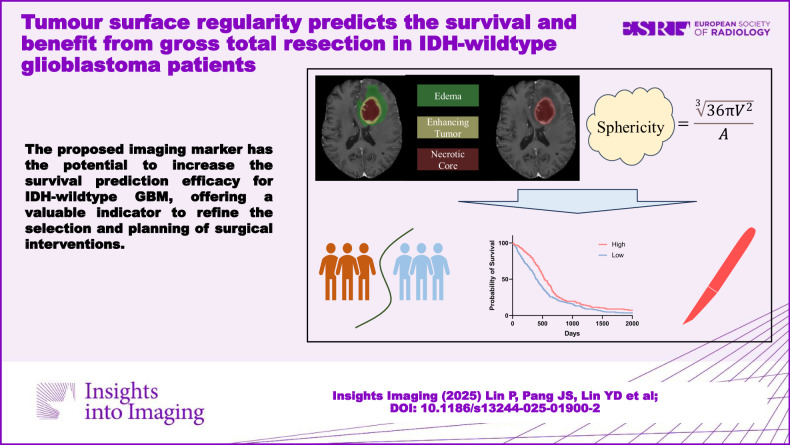

## Introduction

Glioblastoma (GBM), the most common primary brain tumour in adults, represents approximately 48% of all brain tumours [1]. Despite some progress in the treatment of GBM, the 5-year survival rates are still less than 10% [2, 3]. Maximal and safe resection is the guiding principle for GBM therapy [4, 5]. Recent studies have provided evidence that maximising the extent of resection (EOR) improves survival outcomes irrespective of the molecular status [6, 7]. However, gross total resection (GTR) is not always feasible, especially when dealing with tumours located in functional regions [7, 8]. Therefore, the selection of precise and individualised treatment measures is crucial for the prognosis and quality of life of patients. Drexler R et al [9] reported that RTK I and RTK II GBM patients could receive a survival benefit from GTR but not from the MES subclass. Medical imaging indicators have good clinical value owing to their noninvasive and repeatable nature, but they have not yet been utilised effectively.

Radiomics is a technique that extracts high-dimensional features from medical images to enable quantitative analysis of tumour phenotypes [10, 11]. Radiomics technology has been widely used in GBM patients. Radiomics algorithms can assist in characterising core signalling pathways and potentially provide guidance for targeted therapy in IDH-wildtype GBM [12]. Radiomics is an effective tool for the prognostic analysis of GBM, and survival-related features are associated with various molecular features of tumours, enhancing the interpretability of the technology [13, 14]. However, radiomics often requires deep learning or machine learning algorithms to build models, which may have limitations in terms of the interpretability of features and clinical translation. Interpretable radiomic features need further research to promote their clinical application. Some morphological features play important roles in evaluating tumour heterogeneity. A previous study reported that the surface regularity parameter of GBM patients was an indicator of survival and was helpful in predicting surgical response [15].

Here, we aimed to explore the prognostic value of sphericity in IDH-wildtype GBM patients. Patients can be classified into different subgroups based on sphericity, with significant survival differences. We show that patients with surface irregularities receive significant benefits from GTR, whereas patients with surface-regular tumours do not.

## Materials and methods

### Study samples

This study protocol involving the use of deidentified data was approved by the Institutional Review Board of our hospital. All patient-derived clinical, imaging, and genomic data analysed in this study were publicly available and previously deidentified in The Cancer Imaging Archive (TCIA) database [16]: University of California San Francisco (UCSF) [17] and University of Pennsylvania (UPenn) [18].

**Fig. 1:**
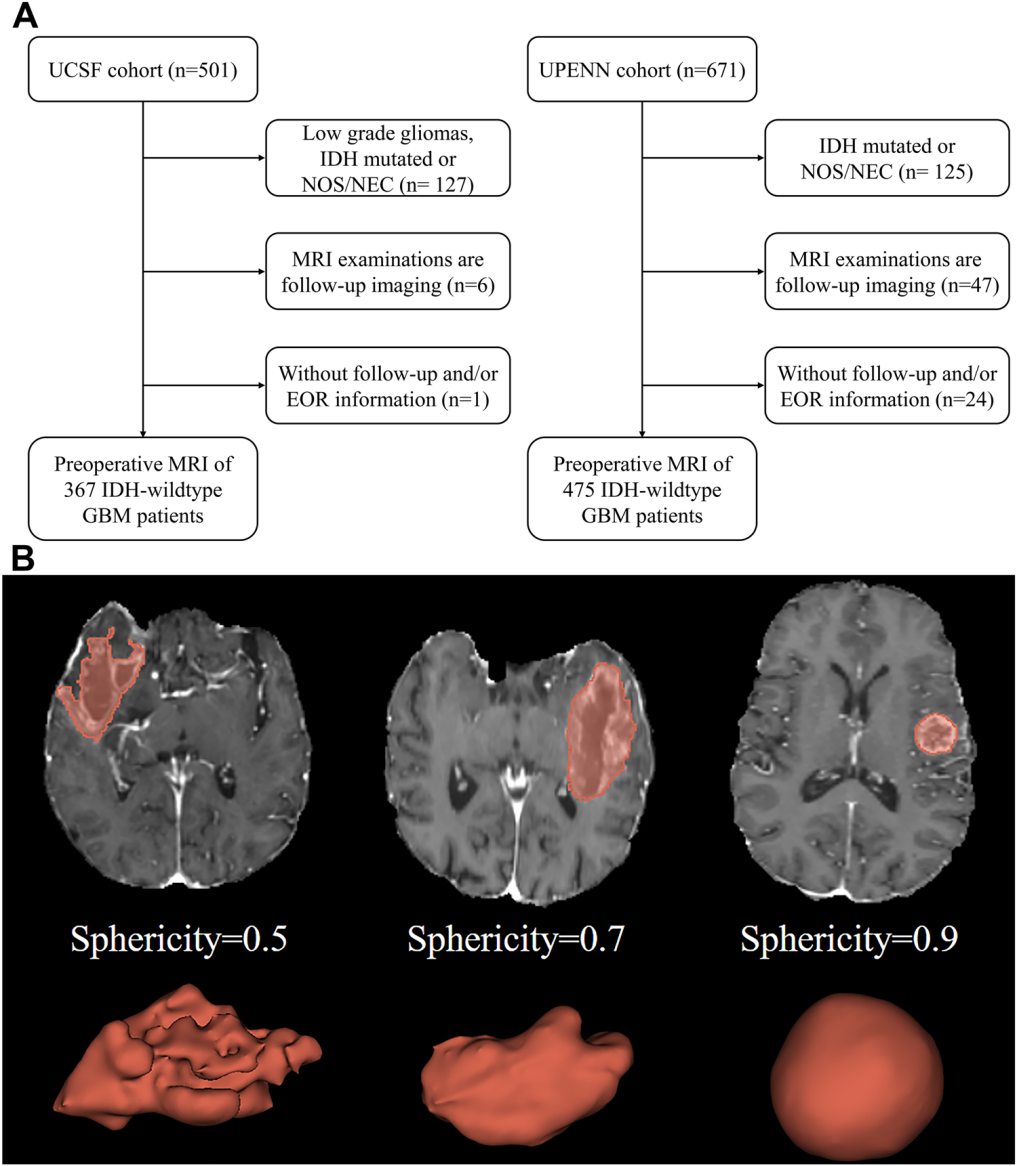
Flowchart and examples of three-dimensional reconstruction and sphericity calculation results. **A** Flowchart showing the patient inclusion process protocol for each cohort. The two datasets used are publicly available in The Cancer Imaging Archive database. **B** Sphericity measures the degree of roundness of the tumour region in comparison to that of a sphere. The larger the sphericity measurement value is, the more regularity it represents. A sphericity equal to 1 indicates a perfect sphere. 3D surface representation of the tumours of 3 patients with different sphericity scores: 0.5, 0.7, and 0.9

A total of 842 patients from the UCSF and UPenn cohorts were included to explore the prognostic value and GTR response prediction value of sphericity. Patients who met the following criteria were included: (1) had IDH-wildtype GBM, (2) had baseline MRI data available before treatment, and (3) had available follow-up and EOR information for OS. The exclusion criteria were as follows: (1) MRI examinations were performed during follow-up, (2) patients whose IDH mutation and IDH mutation information was not otherwise specified/not otherwise classified (NOS/NEC), and (3) follow-up and/or EOR information for OS was not available (Fig. 1A). The criterion for GTR was the absence of any residual enhancing tumour on these postoperative MRIs, which is a standard measure in neurosurgical oncology for assessing the completeness of resection.

### MRI acquisition parameters

MRI examination images and segmentation files are available in the TCIA database. Preoperative multimodel MRI scans include four MRI models: T1-weighted (T1), postcontrast T1 (T1C), T2-weighted (T2), and T2 fluid attenuated inversion recovery (T2-FLAIR). Gadolinium-based contrast agents were used in contrast-enhanced examinations. For the UCSF dataset, all MRI scans were obtained via a 3.0-Tesla scanner (GE Healthcare, Wisconsin, USA) and a dedicated 8-channel head coil. For the UPENN dataset, all MRI scans were obtained via a 3.0-Tesla scanner (Siemens, Erlangen, Germany) with a 12-channel phased array coil.

### Preprocessing and segmentation

All multimode MR images were reoriented to the left-posterior-superior region and coregistered to the same T1 anatomic template. Then, they were resampled to a spatial resolution of 1 × 1 × 1 mm^3^. The images were then skull-stripped for tumoural subregion segmentation. Automatic segmentation of three major tumoural subregions, namely, the enhancing tumour (ET), necrotic tumour core (NCR), and peritumoural oedema (ED) subregions, was performed via machine learning-based brain tumour segmentation. Compared with those of the T1 model, the NCR and ET subregions can be distinguished on the basis of areas that appear hyperintense and hypointense on T1-Gd, respectively. Biologically, enhancement of a tumour signifies the presence of areas where there is leakage of contrast agent due to a disrupted blood‒brain barrier, which is frequently observed in high-grade gliomas. The ED subregion is a hyperintense area on T2-FLAIR images.

Briefly, the patients’ coregistered and skull-stripped structural multimodel MR images underwent automated segmentation. For the UCSF dataset, automated segmentation via an ensemble model consisting of international brain tumour segmentation (BraTS) is the best segmentation algorithm. The segmentations were then manually corrected by two expert reviewers [17]. For the UPENN dataset, automated segmentation processes were also performed on the basis of the BraTS challenge top-ranked deep learning algorithms, and then the label fusion technique was used to combine the results of the different algorithms [18].

### Sphericity calculation

Based on the tumour images and corresponding segmentation files, radiomic features were extracted from these datasets via PyRadiomics version 3.0.1 software [19]. Sphericity is one of the shape features that is calculated from the three-dimensional ROI. The value of sphericity ranges from 0 to 1, where a value of 1 indicates a perfect sphere. Hence, sphericity is a measure of the roundness of the shape of the tumour region relative to a sphere. The sphericity was calculated via the following formula: ${Sphericity}=\frac{\root 3\; \of {36{{{\rm{\pi }}}}{V}^{2}}}{A}$, where V is the volume of the mesh in mm^3^ and A is the surface area of the mesh in mm^2^. In accordance with previous publications [15], the “tumour core” (TC) region containing NCR and ET labels was used for sphericity calculations and further analysis.

### Statistical analysis

All the statistical analyses were performed with R software (version 4.2.2). OS was the endpoint in this study. An OS event was identified as death from any cause. Kaplan–Meier (K-M) plots with log-rank tests were analysed to identify survival differences. The “survminer” software was used to identify the optimal cut-off point for separating patients into separate groups with distinct prognoses. The optimal cut-off for sphericity was determined by the lowest log-rank *p*-value, and each group was required to have at least 25% of the total sample size for grouping. Prognosis associated with sphericity was quantified using hazard ratios (HRs) and 95% confidence intervals (CIs). Multivariate Cox analyses were performed to determine whether the sphericity-based survival subgroup algorithm was an independent prognostic factor. In the enter method, all variables are entered into the model simultaneously. The restricted mean survival time (RMST) model was used to analyse the survival benefit difference via the “survRM2” package in R software. Statistical significance was identified with *p*-values < 0.05 unless otherwise noted.

## Results

### Patient characteristics

The discovery cohort (UCSF cohort) included 367 patients (median age, 62.0 years [IQR, 54.5–70.5 years]). The validation cohort (UPenn) included 475 patients (median age, 63.6 years [IQR, 56.2–71.3 years]). The baseline clinicopathological and molecular features of the included patients are summarised in Table 1.

**Table 1:** Patient characteristics

Characteristic	UCSF-PDGM (*n* = 367)	UPenn-GBM (*n* = 475)
Age		
Median (IQR)	62.0 (54.5–70.5)	63.6 (56.2–71.3)
Sex		
Female	149 (40.6)	188 (39.6)
Male	218 (59.4)	287 (60.4)
MGMT		
Unmethylated	145 (39.5)	141 (29.7)
Methylated	203 (55.3)	99 (20.8)
Indeterminate or N/A	19 (5.2)	235 (49.5)
KPS score		
≤ 80	–	31 (6.5)
90–100	–	41 (8.6)
N/A	367 (100)	403 (84.8)
GTR		
Yes	214 (58.3)	292 (61.5)
No	153 (41.7)	183 (38.5)

*GTR* gross total resection, *IQR* interquartile range, *KPS* Karnofsky Performance Scale, *MGMT* O-6-methylguanine-DNA methyltransferase, *N/A* not applicable

### Prognostic value of sphericity

Sphericity was extracted from the TC mask labels for analysis. Sphericity can be used to quantify the surface regularity of tumours (Fig. 1B). Patients were divided into different subgroups according to their sphericity score. The thresholds that best divided the patients into significant subgroups were determined.

**Fig. 2:**
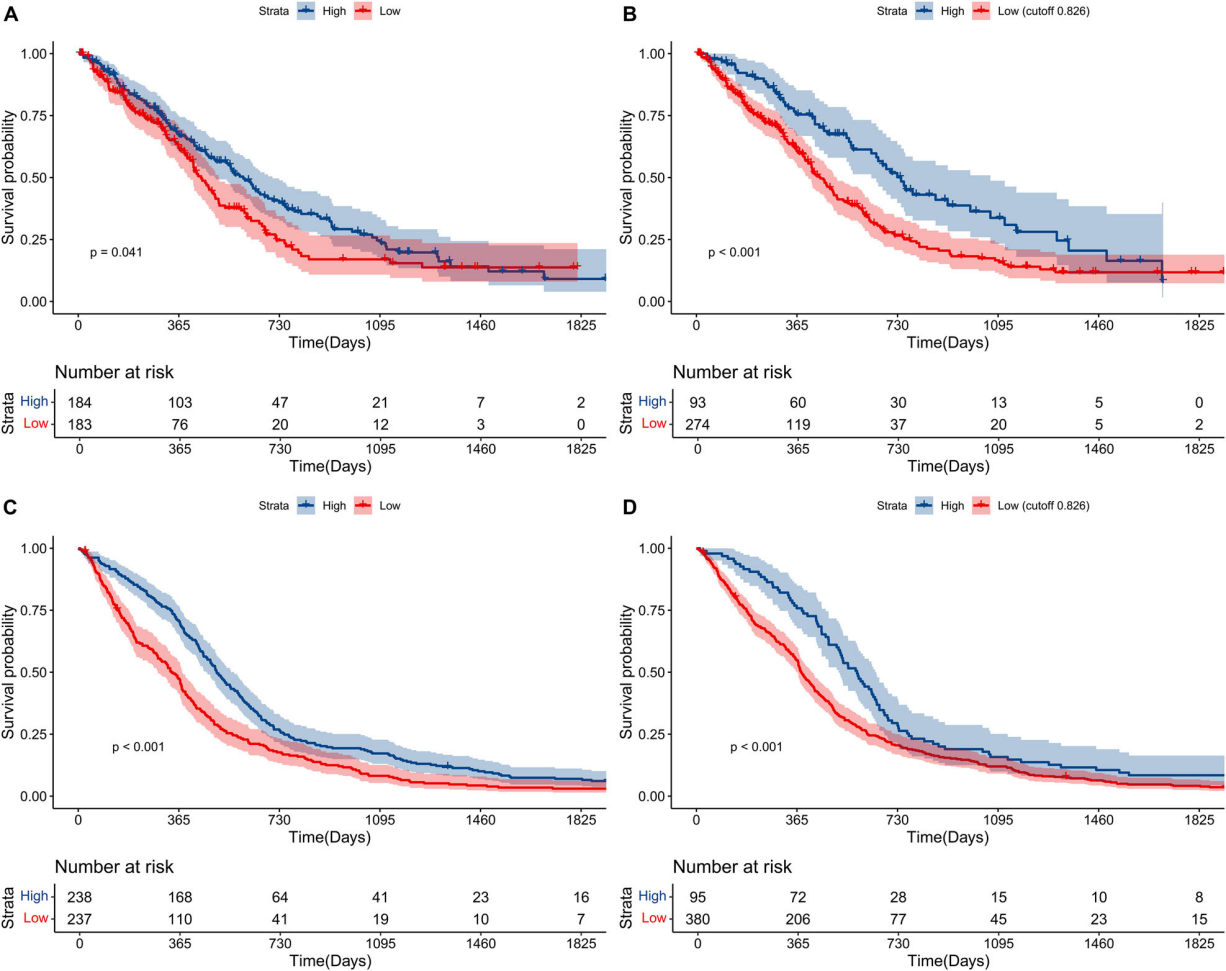
Kaplan‒Meier plots for the groups with different surface regularity. Tumours with regular surfaces were significantly correlated with improved OS in the discovery cohort based on median value (**A**) and optimal cut-off value (0.826) (**B**). In the validation cohort, GBM patient data also revealed that a regular surface was significantly correlated with improved OS on the basis of the median value (**C**) and optimal cut-off value (0.826) determined in the discovery cohort (**D**)

In the discovery cohort (UCSF cohort), the optimal cut-off of sphericity was determined to be 0.826. Based on the median value (Fig. 2A) and optimal cut-off (Fig. 2B) of sphericity, K‒M plots revealed that patients with high sphericity were significantly associated with longer OS in the discovery cohort. In the validation cohort, patients in the surface regularity subgroup also had superior OS than patients in the surface irregularity subgroup, whether distinguished by the median value (Fig. 2C) or the optimal value (Fig. 2D) determined in the discovery cohort.

Univariate Cox analysis revealed that sphericity achieved statistically significant OS differences in the discovery cohort (HR = 1.73, 95% CI: 1.27–2.37, *p* < 0.001) and the validation cohort (HR = 1.47, 95% CI: 1.17–1.86, *p* < 0.001). Multivariate Cox analysis further confirmed that the sphericity-based survival subgroup was an independent prognostic factor for OS in GBM patients after adjusting for clinicopathological and molecular parameters in both the discovery cohort (HR = 1.45, 95% CI: 1.06–1.99, *p* = 0.022) and the validation cohort (HR = 1.38, 95% CI: 1.09–1.74, *p* = 0.007) (Table 2).

**Table 2:** Univariable and multivariable Cox analysis of sphericity-based survival subgroups in discovery and validation datasets

Parameters	Univariable analysis		Multivariable analysis	
	HR (95% CI)	*p-v*alue	HR (95% CI)	*p-v*alue
Discovery cohort (UCSF cohort)				
Age (continues)	1.03 (1.02–1.05)	< 0.001	1.04 (1.03–1.05)	< 0.001
Sex				
Female (*n* = 149)	1 (Reference)	–	1 (Reference)	–
Male (*n* = 218)	1.15 (0.88–1.50)	0.317	1.20 (0.91–1.58)	0.194
MGMT				
Unmethylated (*n* = 145)	1 (Reference)	0.043	1 (Reference)	0.164
Methylated (*n* = 203)	0.73 (0.55–0.96)	0.025	0.77 (0.58–1.01)	0.062
N/A (*n* = 19)	1.15 (0.64–2.06)	0.647	0.95 (0.52–1.73)	0.867
EOR				
No-GTR (*n* = 153)	1 (Reference)	< 0.001	1 (Reference)	< 0.001
GTR (*n* = 214)	0.50 (0.38–0.65)	< 0.001	0.49 (0.37–0.65)	< 0.001
Sphericity subgroup				
High (*n* = 93)	1 (Reference)	–	1 (Reference)	–
Low (*n* = 274)	1.73 (1.27–2.37)	< 0.001	1.45 (1.06–1.99)	0.022
Validation cohort (UPENN cohort)				
Age (continues)	1.02 (1.01–1.03)	< 0.001	1.02 (1.01–1.03)	< 0.001
Sex				
Female (*n* = 188)	1 (Reference)	–	1 (Reference)	–
Male (*n* = 287)	1.02 (0.84–1.23)	0.871	0.99 (0.82–1.19)	0.904
MGMT				
Unmethylated (*n* = 141)	1 (Reference)	< 0.001	1 (Reference)	< 0.001
Methylated (*n* = 99)	0.51 (0.39–0.66)	< 0.001	0.41 (0.31–0.54)	< 0.001
N/A or indeterminate (*n* = 235)	0.72 (0.58–0.89)	0.003	0.70 (0.56–0.86)	0.001
GTR				
No (*n* = 183)	1 (Reference)	–	1 (Reference)	–
Yes (*n* = 292)	0.65 (0.54–0.78)	< 0.001	0.69 (0.56–0.84)	< 0.001
Sphericity				
High (*n* = 95)	1 (Reference)	–	1 (Reference)	–
Low (*n* = 380)	1.47 (1.17–1.86)	< 0.001	1.38 (1.09–1.74)	0.007

*GTR* gross total resection, *HR* hazard ratio, *MGMT* O-6-methylguanine-DNA methyltransferase, *NA* not applicable

**Fig. 3:**
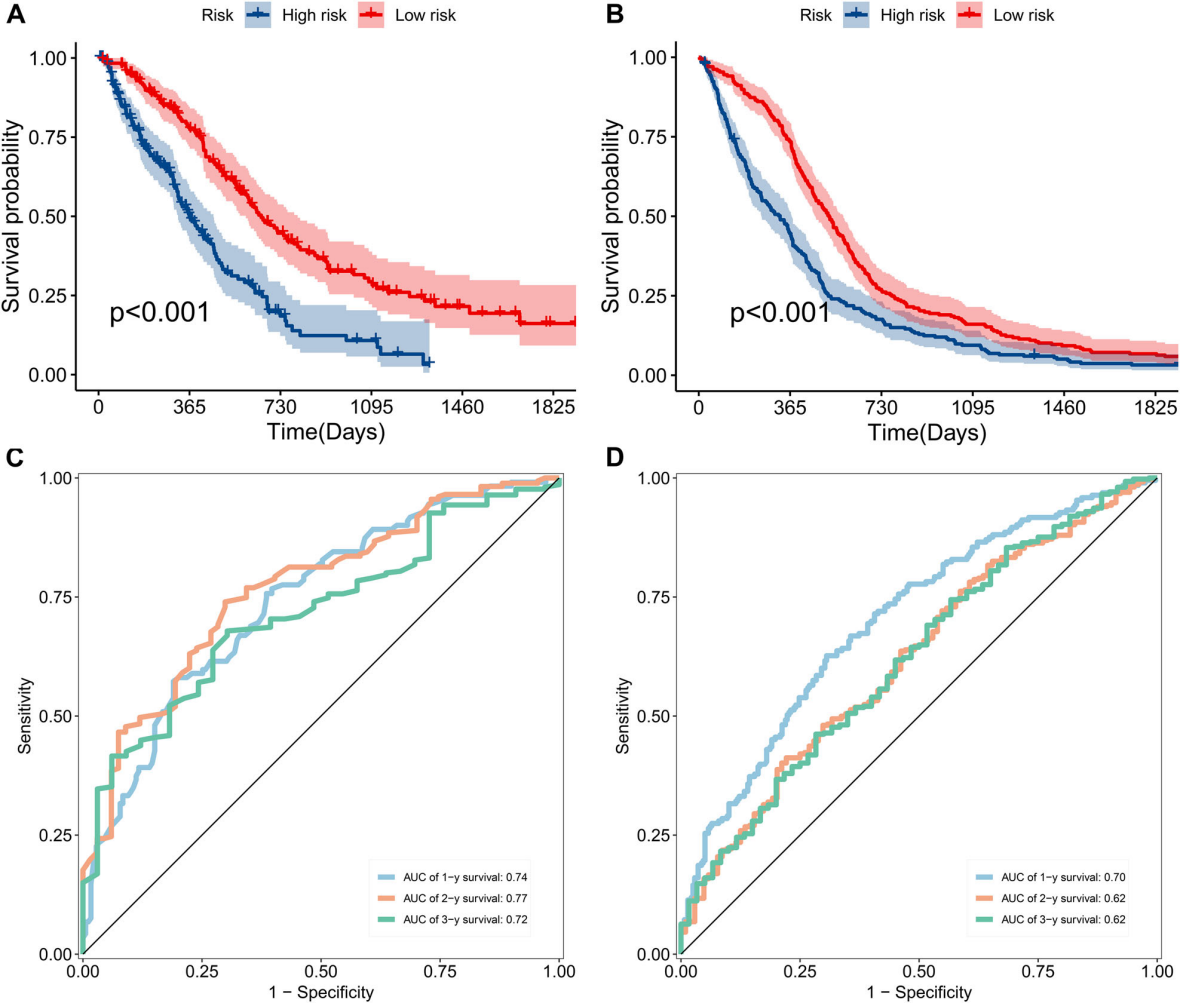
The prognostic value of the risk model. Kaplan–Meier (K-M) plots show that patients in the high-risk subgroup had shorter OS than patients in the low-risk subgroup did based on the median value of the risk model in the discovery (**A**) and validation cohorts (**B**). The time-dependent receiver operating characteristic (ROC) curves show that the survival risk model is effective for survival prediction in the discovery (**C**) and validation (**D**) cohorts

In the discovery cohort, multivariate analysis suggested that age, EOR and surface regularity were independent prognostic factors, suggesting their complementary value. To further improve the accuracy of prognosis prediction, we combined age, the EOR, and the surface regularity score to fit a Cox proportional hazards regression model as follows: score = 0.036 * age + 0.385 * surface regularity subgroup − 0.738 * EOR. Based on the median risk score of the integrated risk model, patients in the high-risk group had poorer OS than patients in the low-risk group did (HR = 2.16, 95% CI: 1.63–2.85, *p* < 0.001, Fig. 3A) and those in the validation cohort did (HR = 1.59, 95% CI: 1.32–1.92, *p* < 0.001, Fig. 3B). Time-dependent ROC curves for the training (Fig. 3C) and validation (Fig. 3D) cohorts revealed that this risk model was a moderate algorithm for prognosis prediction.

For the discovery cohort, 53, 37, and 3 samples in the high sphericity group were methylated, unannotated and unavailable for MGMT, respectively (*n* = 93). In the low sphericity group (*n* = 274), 150, 108 and 16 samples were methylated, unmethylated and unavailable for MGMT, respectively. No significant difference in the proportion of methylated MGMT was observed between different sphericity statuses (*p* > 0.05). For the validation cohort, 16, 29, and 50 samples in the high sphericity group were methylated, unannotated and unavailable for MGMT, respectively (*n* = 95). In the low sphericity group (*n* = 380), 83, 112, and 185 samples were methylated, unmethylated and unavailable for MGMT, respectively. No significant difference in the proportion of methylated MGMT was observed between different sphericity statuses (*p* > 0.05).

### Sphericity and GTR survival benefit

**Fig. 4:**
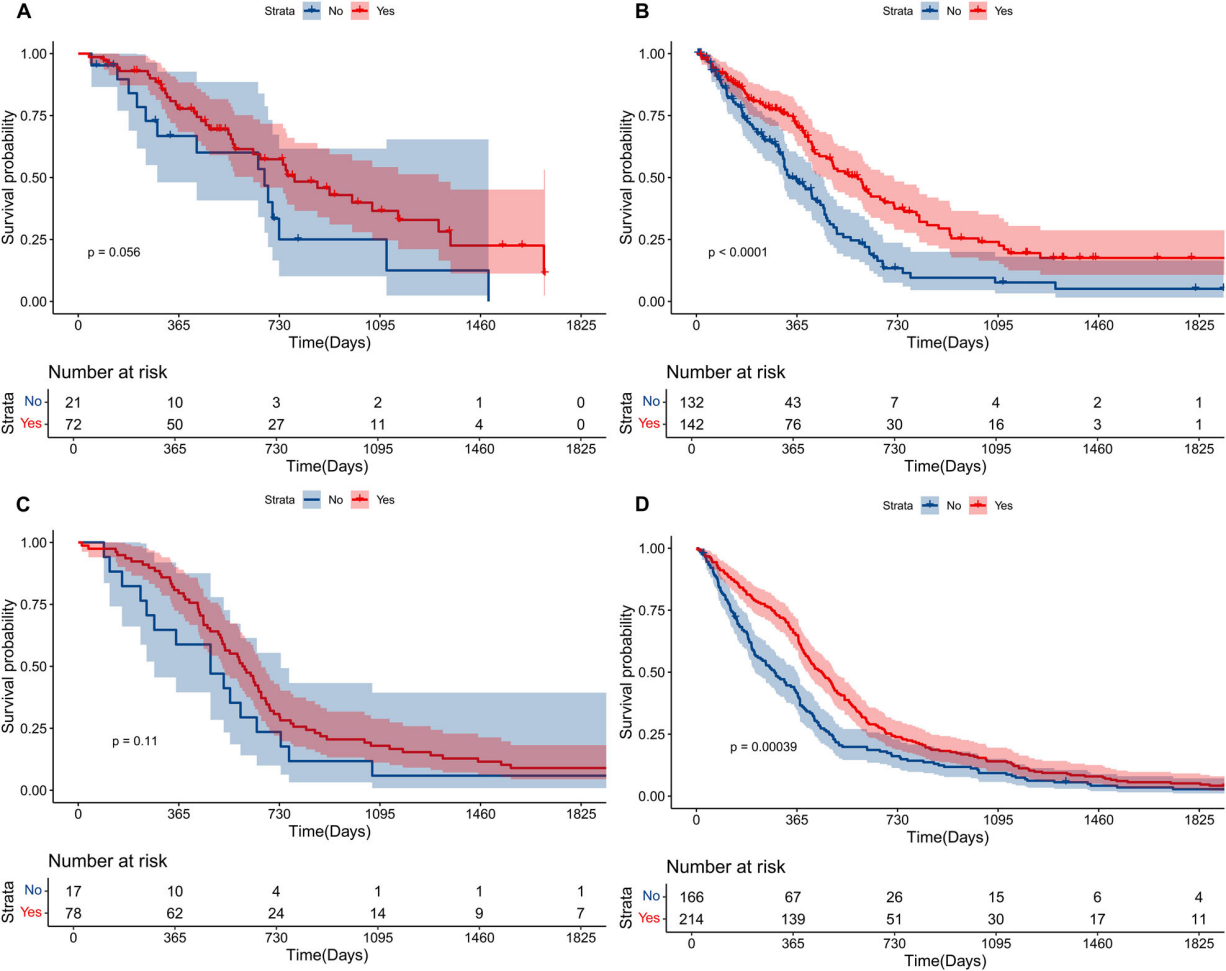
The prognostic value of gross tumour resection (GTR) varies among patients with different surface regularity features. The Kaplan–Meier (K-M) plots show no significant improvement in OS in patients with surface-regular tumours treated with GTR (**A**), whereas GTR improved OS in those with surface-irregular tumours (**B**) in the discovery cohort. The K-M plots show no significant improvement in OS in patients with surface-regular tumours treated with GTR (**C**), whereas GTR improved OS in those with surface-irregular tumours (**D**) in the validation cohort

We investigated differences in OS benefit from GTR between the surface regularity subgroups of patients. We found that, in the discovery cohort, there was no significant OS benefit in patients with surface-regular tumours (high sphericity) (HR: 0.56, 95% CI: 0.27–1.15, *p* = 0.056; Fig. 4A), whereas patients with irregular tumours (low sphericity) received a substantial OS benefit from GTR (HR: 0.55, 95% CI: 0.40–0.75, *p* < 0.001; Fig. 4B). In the validation cohort, we also observed that patients with surface-regular tumours did not receive an OS benefit from GTR (HR: 0.66, 95% CI: 0.36–1.21, *p* = 0.112; Fig. 4C) compared with patients with surface-irregular tumours (HR = 0.69, 95% CI: 0.56–0.86, *p* < 0.001; Fig. 4D).

Our analysis revealed an increase in RMST for patients who received GTR compared with those who received non-GTR in the low sphericity subgroup over a follow-up of 1 year, 3 years and truncation (Table 3). At the truncation time point, for surface irregularity tumours, the RMST ratio between the GTR and no-GTR groups was 1.64 (95% CI, 1.26–2.13; *p* < 0.001) in the discovery cohort and 1.52 (95% CI, 1.17–1.97; *p* = 0.002) in the validation cohort. For surface regularity tumours, the RMST ratio between the GTR and no-GTR groups was 1.37 (95% CI, 0.95–1.98; *p* = 0.093) in the discovery dataset and 1.65 (95% CI, 0.99–2.74; *p* = 0.054) in the validation cohort.

**Table 3:** RMST ratio between GTR and non-GTR groups in different sphericity subgroups

		High sphericity		Low sphericity	
		RMST ratio (95% CI)	*p*-value	RMST ratio (95% CI)	*p*-value
Discovery cohort (UCSF cohort)	1-year	1.10 (0.95–1.27)	0.189	1.13 (1.03–1.25)	0.009
	3-year	1.22 (0.91–1.65)	0.187	1.47 (1.23–1.76)	< 0.001
	Truncation	1.37 (0.95–1.98)	0.093	1.64 (1.26–2.13)	< 0.001
Validation cohort (UPENN cohort)	1-year	1.12 (0.96–1.31)	0.142	1.23 (1.12–1.35)	< 0.001
	3-year	1.27 (0.94–1.71)	0.127	1.37 (1.17–1.60)	< 0.001
	Truncation	1.65 (0.99–2.74)	0.054	1.52 (1.17–1.97)	0.002

*RMST* restricted mean survival time, *GTR* gross total resection

## Discussion

Radiomics can capture tumour heterogeneity based on medical imaging phenotypes. However, numerous limitations have been identified, leading to obstacles for its clinical application. These limitations include a lack of generalisability and challenges in identifying reliable and practical clinical biomarkers [20, 21]. Currently, many machine learning or deep learning algorithms are used for radiomics model development. These algorithms require reproducibility of classifier development methods and complex statistical validation and instead ignore the clinical value of the feature itself. Here, we determined that the surface regularity parameter sphericity of IDH-wildtype GBM patients was an independent indicator of OS. Furthermore, we found that subgroups determined by sphericity could identify patients who may receive an OS benefit from GTR.

The first strength of our study is the simplicity of sphericity, which can predict OS in IDH-wildtype GBM patients via routine clinical scans without the need for advanced neuroimaging data. The findings of previous studies revealed several imaging phenotypes for OS prediction in GBM patients. Some excellent studies have provided artificial intelligence-based technology for survival prediction in GBM patients [22, 23], which has shown that quantitative medical images have the potential to accurately evaluate tumour heterogeneity. Radiomics has shown great promise in differentiating the clinical outcomes of GBM patients [24, 25]. In our multivariate analysis, age, EOR and sphericity-based subgroups were found to be independent factors for GBM patient OS. The noninvasive prognostic model showed moderate prognosis prediction performance. Without losing performance, our model has the advantages of simplicity and interpretability. Harsh tumour microenvironment conditions may cause tumours to grow with morphological invasiveness [26]. These conditions mainly include hypoxia and a heterogeneous extracellular matrix, which is a key feature of the infiltrative phenotypes of GBM [27, 28]. These theories may explain the prognostic value of sphericity.

Another result that should be noted is that patients with low sphericity GBM are more likely to receive survival benefit from GTR. Notably, a previous excellent study also showed that the surface regularity of GBM is a survival predictor [15]. They reported that tumours with regular surfaces were closely correlated with longer OS than tumours with irregular surfaces were. The multivariate analysis revealed that age and surface regularity were significant variables. These findings are consistent with our findings. However, they reported that patients with surface-regular tumours benefit substantially from GTR, whereas patients with surface-irregular tumours do not. This finding contradicts what we have observed previously, which could be due to different threshold selections or heterogeneity among different datasets. The 2021 WHO classification classified adult-type diffuse gliomas into the following: (1) astrocytoma, IDH-mutant; (2) oligodendroglioma, IDH-mutant, and 1p/19q-codeleted; and (3) glioblastoma, IDH-wildtype [29]. Therefore, our study included only IDH-wildtype GBM for analysis. Therefore, the cut-off for OS risk stratification may differ owing to different molecular statuses, and further validation with larger samples is needed to investigate the relationship between sphericity and GTR in the future. A recent study revealed that newly diagnosed and recurrent GBM patients with DNA methylation subclasses RTK I and RTK II would receive a survival benefit from the maximised extent of resection. However, patients with the mesenchymal subtype do not benefit from this approach [9]. With the increasing comprehension of GBM patients’ imaging and molecular characteristics, stratifying patients based on their suitability for GTR, particularly in cases of GBM involving functional areas or recurrent GBM, has potential.

In our study, several limitations should be discussed. First, this retrospective study included patients from different centres rather than from a prospective randomised clinical trial, and the collected clinicopathological or molecular parameters may have heterogeneity depending on different standards. To validate the prognostic and predictive value of sphericity, further prospective analyses should be performed. Second, no data for adjuvant chemotherapy or other treatments were available. The influence of different treatment approaches, such as adjuvant therapy, should be validated in future studies. Third, future studies should integrate multiomics molecular information, which could provide a complete molecular landscape for imaging phenotypes in IDH-wildtype GBM.

In conclusion, our study revealed that high sphericity in IDH-wildtype GBM patients was associated with longer OS. Compared with patients with high sphericity, patients with low sphericity benefit from GTR in terms of OS. These findings validate sphericity as a reliable outcome and surgical response predictor that may be considered in clinical practice. However, the optimal cut-off value for sphericity should be further validated in the future.

## Data Availability

The images and segmentation files reported in this study can be obtained from The Cancer Imaging Archive database using the accession numbers UCSF-PDGM [30] and UPENN-GBM [31].

## Abbreviations

ED: Peritumoural oedema
EOR: Extent of resection
ET: Enhancing tumour
GTR: Gross total resection
K‒M: Kaplan‒Meier
MGMT: O-6-methylguanine-DNA methyltransferase
NCR: Necrotic tumour core
OS: Overall survival
TCIA: The Cancer Imaging Archive
